# Compounding social-ecological crises drive spatial mobility and land abandonment in Morocco’s High Atlas

**DOI:** 10.1007/s13280-025-02323-5

**Published:** 2025-12-19

**Authors:** Laura Marlene Kmoch, Aimad Bou-Lahriss, Rachid Ait Babahmad, Tobias Plieninger

**Affiliations:** 1 Chair of Social-Ecological Interactions in Agricultural Systems, University of Kassel and University of Göttingen, Platz der Göttinger Sieben 5, 37073 Göttingen, Germany; 2 Ouarzazate, Morocco; 3 Semlalia Faculty of Sciences, Water Sciences, Microbial Biotechnology and Sustainability of Natural Resources Laboratory, UCA, Cadi Ayyad University, Mark Herbarium, Bd. Prince My Abdellah, B.P. 2390, 40000 Marrakech, Morocco; 4 Moroccan Biodiversity and Livelihoods Association, 1 Rue Houcima, Marrakech, Morocco

**Keywords:** Drought, Dryland systems, Environmental migration, Land attachment, Land-use decision-making, Livelihood transformations

## Abstract

Drought and water insecurity are escalating crises that shape dryland farmers’ mobility and land abandonment decisions. In this study, we appraised the causes of such decisions through 75 qualitative interviews and six group discussions with residents of rural Amazigh communities in Morocco’s High Atlas. We identified a multi-faceted crises nexus, where water scarcity exacerbates economic and value-related crises and personal insecurities, leading to a crisis of confidence in traditional rural lifeways. Increasingly multi-local household structures and intersectional attributes mediate actors’ experiences, as illustrated by a typology we present. While some actors experienced immobility as a hardship, other desired to stay, underscoring the continued importance of *tamazirt*—an Amazigh concept linking land and home—as a promising anchor for land-futures thinking. By addressing evidence gaps on the role of crises and intersectional characteristics in dryland decision-making, our study offers insights for rural development planning and policy in the southern Mediterranean.

**Supplementary Information:**

The online version contains supplementary material available at 10.1007/s13280-025-02323-5.

## Introduction

Drylands are home to almost a third of the world’s population, and the number of dryland dwellers continues to rise. At the same time, the severity of water security threats affecting them—such as drought and irrigation-driven groundwater decline—is also growing (Jasechko et al. 2024; Toreti et al. 2024; UNESCO 2024; Vincente-Serrano et al. 2024). For instance, prolonged droughts in the Mediterranean have recently disrupted Syrian farmers’ harvests (Dinc and Eklund 2024), cereal yields in southern Europe (Rossi et al. 2023), and the production of olives in Italy, Morocco, and Spain (Blanco et al. 2024; Wang et al. 2024). Worsening the already challenging life circumstances of many dryland farmers, these threats have induced a severe global water crisis (Grafton et al. 2024; UNESCO 2024). In this context, diverse human mobilities—including outmigration and deliberate or involuntary immobility—can be interpreted as adaptive strategies of dryland farmers in response to water scarcity and climate-related hazards, which often interact with other crises that exacerbate vulnerable people’s livelihoods stress (Afifi et al. 2016; Boas et al. 2022; UNESCO 2024; Borgomeo and Jägerskog 2025; Malik and Ford 2025).

In addition to spatial mobility decisions, the restructuring of rural livelihoods in a crisis context is also often intertwined with land-change dynamics, including agricultural land abandonment (Afifi et al. 2016; Meyfroidt et al. 2022; Piquer-Rodríguez et al. 2023). Research on land abandonment has gained traction since the 2000s, with studies addressing its drivers, processes, and impacts at multiple spatial scales (Munroe et al. 2013; Subedi et al. 2022; Zhou et al. 2024). This includes debates on trade-offs, response options, and potential benefits, particularly regarding biodiversity (Daskalova and Kamp 2023). However, evidence on land abandonment in North Africa remains strikingly limited, as do studies that disentangle mobility and land abandonment decisions in drought affected settings—though notable exceptions exist (Quintas-Soriano et al. 2022; Subedi et al. 2022; Dinc and Eklund 2024). This is concerning, as land abandonment is “the product of complex, age-old interactions between people and nature” rather than “a purely social or ecological phenomenon” (Daskalova and Kamp 2023, 582), with critical implications for rural development planning. Thus, it calls for integrated social-ecological research, and heightened scrutiny of its multiple facets, especially in hitherto understudied regions (Munroe et al. 2013; Subedi et al. 2022).

In Morocco—where water scarcity receives increasing policy attention—droughts severely affect farmers’ cereal and tree-crop yields, the nation’s food import dependence, and agricultural sector employment (Alfani et al. 2024; Kmoch et al. 2024). The latest prolonged drought and dwindling water reserves have brought heightened attention to many farmers’ perilous overreliance on irrigation water, which often goes hand in hand with the production of export crops (Taheripour et al. 2020; Fico 2024). Studies on water insecurity and environmental changes have linked migration to adaptation, but caution that the connection is often fuzzy (Van Praag et al. 2021; Kmoch et al. 2024). Structural migration research and long-term ethnography further emphasise that shifting mobility patterns are rooted in broader societal transformations (Berriane et al. 2021), including the gradual integration of Morocco’s mountain farmers into capitalist production relations (Crawford 2008). However, evidence on the impacts of recent droughts on North Africa’s agricultural labour force remains scarce (Alfani et al. 2024), particularly regarding Moroccan farmers’ intertwined mobility and land abandonment decisions—except for earlier system dynamics research on terrace landscapes in the Anti-Atlas and a recent study in Souss-Massa (Boselli et al. 2020; Ferreira Fernandes et al. 2024).

Against this backdrop, we untangle key strands of an unresolved phenomenological puzzle: In late 2021, we spoke to interviewees in a drought-plagued mountain area. Our respondents in Morocco’s High Atlas recounted how years with low rainfall and water scarcity had hampered their harvests, pushing their production systems and livelihoods to the brink (Kmoch et al. 2024). Yet, their plight entailed more than a water crisis: They experienced profound social-ecological transformations, as ever more residents appeared to abandon their farmland in favour of out-migration, urban lifestyles, and off-farm work. What remained unclear, however, was to what extent these decisions were driven by drought, vis-à-vis other factors and crises experiences, and what they implied for the future of agricultural livelihoods and local residents’ attachment to land. Our aim with the present study was to understand the roots and consequences of dryland farmers’ mobility and land-abandonment decisions in a protracted drought-crisis context.

Four research questions structured our inquiry:

1. What proximate causes motivate residents’ spatial mobility patterns?
2. What underlying causes shape their mobility and land-abandonment decisions?
3. How do diverse household constellations and individuals’ intersectional characteristics mediate mobility and land abandonment decisions?
4. How do mobility and land abandonment decisions shape residents’ future aspirations and affective ties to land?

## Materials and methods

### Analytical framework

*Sustainable livelihoods thinking*, centred on rural people’s diverse assets, activities, and relations for making a living (Natarajan et al. 2022), served as the overarching lens for our analysis of mobility and land abandonment decisions. To appraise *spatial mobility patterns*, we distinguish between *domestic and international out-migration, in-migration and rural flight*, and *immobility*, based on the aspirations-capabilities framework and climate mobilities scholarship (de Haas 2021; Boas et al. 2022). *Land abandonment* is a fuzzy and often transitional phenomenon, which here denotes "a decline in farming intensity and complete cessation of farming after a certain time” (Subedi et al. 2022, 6), as this agronomic definition best aligns with our field observations. Finally, drawing on land system science, we distinguished between *proximate* and *underlying causes* of mobility and land abandonment decisions—factors that are either close or distal in the causal chains leading to the studied phenomena (Meyfroidt 2016).

### Study area

**Fig. 1:**
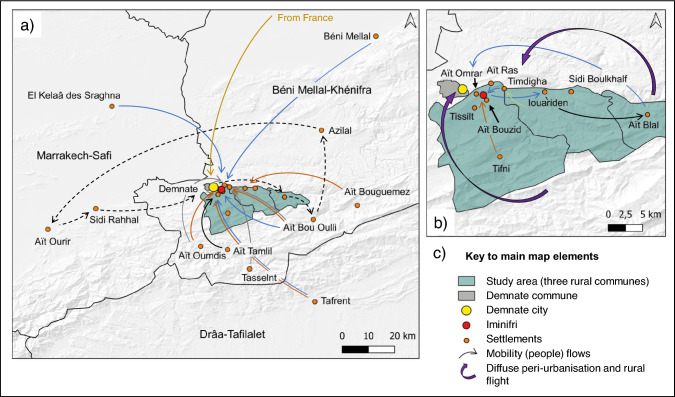
In-migration flows and proximate causes (**a**), and aggregated peri-urbanisation and rural flight (large purple arrows) and local mobility dynamics (**b**). The colours of the slim arrows indicate the proximate causes of respondents’ in-migration and reasons for local mobility: orange = family formation and reestablishment, blue = work related, yellow = return upon retirement, black = investments to pursue entrepreneurial activities, light green = school attendance, grey = unspecified, dashed black = market vendors’ trade-routes

The selection of our study area in Morocco’s Azilal Province was informed by the third author’s established contact with, and intricate knowledge of the region’s rural communities, gained through years of work in a locally active NGO and his personal ties to one of them, paired with an awareness of ongoing yet underexplored outmigration dynamics from the area. We focussed on the eastern outskirts of Demnate (31°43′58″ N, 6°59′42″ W), and three rural communes: Tifni, Sidi Boulkhalf, and Ait Blal (Fig. 1).

**Fig. 2:**
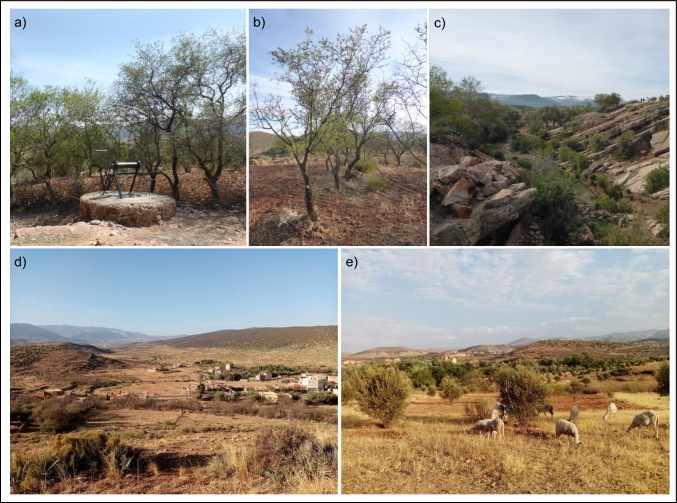
Illustration of drought-affected communities and agricultural landscapes in the study area. **(a)** A well, **(b)** drought affected almond trees, **(c)** a riverbed, **(d)** landscape view, **(e)** livestock among olive trees. The images have been taken by the first author - Laura Marlene Kmoch

The terrain in this area spans an altitudinal gradient from the foothills of the Central High Atlas Massif, located 100 kms east of Marrakech (at ∼ 900 m), to terraced croplands in its steep interior (at ∼ 1600 m). Residents of this region mostly belong to the Amazigh people, who have historically been marginalised, particularly until the late 1990s (Berriane et al. 2021). Similar to other parts of the High Atlas, communities in this area have long lacked basic services, including water, electricity, healthcare, education, communication, and road infrastructure; however, recent improvements have been substantial. Although the region’s cropping systems have remained largely subsistence-oriented, farmers sell surplus grains, vegetables, and tree crops (fruits, olives, and nuts) to commercial intermediaries and regional markets. Primarily market-oriented farms are rare, though groundwater-reliant irrigation is rising. A severe drought crisis substantially diminished farmers’ agricultural yields at the time of our study, and much farmland lay abandoned (Fig. 2). Meanwhile, local off-farm jobs (e.g. in construction, tourism, or trades) were scarce relative to demand.

### Data collection and analysis

Data for this study were generated through qualitative interviews by the first and second authors in October and November 2021. The fieldwork commenced with a key informant interview to generate a list of households residing near Imi-n-ifri in the northernmost part of Tifni Commune. The name of each household head, details about their main occupation, and households’ migration histories were noted. Departing from this list, the second author conducted 75 qualitative interviews and six group discussions (*n* = 3, 3, 6, 6, 8, and 13 participants) with local residents. Participants were selected through purposive sampling, aimed at capturing a wide range of perspectives on our key research themes. Respondents from households with a migration history, residing in villages in the north of Tifni commune were approached first. We then gradually increased the recruitment scope, approaching respondents who were available and willing to engage, and who could provide complementary perspectives to those already captured, e.g. by representing different genders, age groups, (im)mobility trajectories, and occupations (see Table S1). A small subset of respondents in our study were recruited from Sidi Boulkhalf and Ait Blal Communes, to capture livelihood and farming conditions diverging from those in northern Tifni, through better water access but greater remoteness from urban centres and major roads.

All interviews took a conversational tone, but were structured around a set of iteratively developed themes and questions (see Table S2). Not all themes and questions were addressed during all interviews, however, subject to how intensively certain topics had already been covered in previous conversations, and to respondents’ available time and life experiences. A broad spectrum of views was captured until saturation was reached on key interview themes, including respondents’ drought experiences, land-use decisions, family relations, future aspirations, and household members’ occupation and mobility trajectories. The second author’s Amazigh background and fluency in Tamazight (the local Amazigh language), enabled the team to engage with the research participants in a culturally sensitive manner. All interviews were conducted in Tamazight, after informing the relevant Moroccan authorities and obtaining respondents’ informed oral consent. Formal ethical approval by the Central Ethics Committee was not required according to the rules of University of Kassel (University of Kassel 2018).

Respondents’ answers during ten interviews were directly translated by the second author, with the English verbatim fully documented in writing by the first author. The remaining interviews were exclusively conducted by the second author, who took detailed written, partially verbatim notes. Directly following each interview session, both authors collaborated on translating and digitally transcribing and storing the interview notes, from which the English language quotes in this manuscript are drawn. To preserve analytical context, we did not anonymise the interviews during active processing. However, to protect participants’ privacy, access to the transcribed interviews was strictly limited to members of the research team, the data were handled and stored exclusively on password protected university computers, and will be anonymised before archiving.

The qualitative content analysis software MAXQDA (VERBI Software 2024) was used to organise and manually code the English translations of all interviews, employing both deductive and inductive techniques. First, the interviews were read and re-read several times, to assign text segments to broad, deductive codes that aligned with our research themes, i.e. experiences and causes of respondents’ mobility and land abandonment decisions, their effects on individuals and communities, interviewees’ future aspirations, and perceived pathways for rural change. Inductive codes were iteratively developed, to capture sub-themes that were identified throughout the analysis, e.g. capturing aspects such as migrants’ occupations, women’s roles, and the underlying causes of land abandonment.

**Fig. 3:**
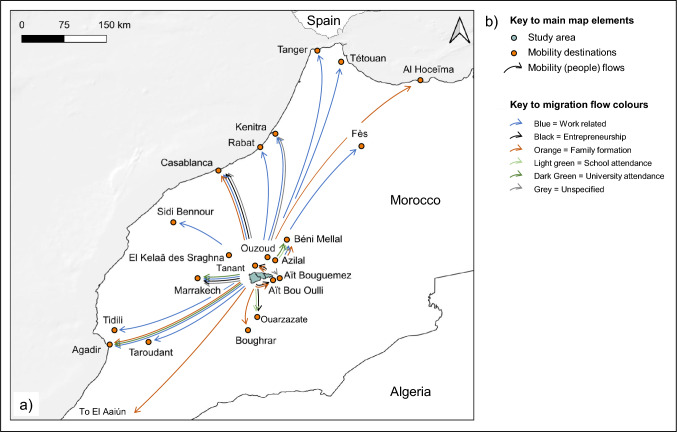
Migration flows to domestic destinations

**Fig. 4:**
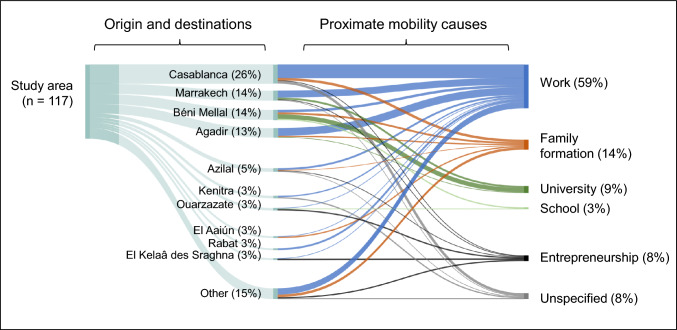
Migration flow shares by domestic destinations and proximate mobility causes

Statements on interviewees’ own mobility reasons and trajectories, and on those of their household members, were organised in a spreadsheet, and sorted by proximate mobility causes and destinations to develop Figs. 1 and 3. Figure 4 was derived from the same data, by calculating percentage shares for each domestic destination and proximate destination cause that were used to generate a Sankey diagram with the SankeyMATIC software (Bogart 2025).

The actor typology in Section “Household constellations and intersectional characteristics” was inductively derived through close reading and iterative comparison of the interview transcripts, in particular respondents’ accounts of their family constellations, life course junctures, crisis experiences, and associated (im)mobility and land-use decisions. Pertinent cases were selected to exemplify the identified categories. The constructed typology thus serves as a heuristic that highlights key tendencies in the data, rather than strictly assigning each respondent, their family constellation, and crises experiences to a distinct category.

The quotations in Tables 1 and 2 were drawn from respondents’ narratives to illustrate key dimensions of social-ecological change and connections to place, which we identified through iteratively coding, synthesising, and reflecting on interviewees’ accounts.

**Table 1:** Social-ecological change dimensions at the root of residents’ spatial mobility and land abandonment decisions

Social-ecological change dimensions	Illustrative quotations	IDs
*Climate and resource crisis*		
Declining water availability	*“30 years ago, the snow was always on the mountains, even during summer time, and the river and valley never got dry. […] Now, the river has less water, and it cannot irrigate all the fields”.*	18
Drought impacts and land devaluation	*“A lot of people had many sheep, donkeys, chicken, rabbits—even during the drought seasons people were always attached to the land. […]. Now everything is changing. The land has no longer the importance—as it was”.*	39
Shifting food habits and convenience expectations	*“People lived the old lifestyle. […]. Mostly growing grains—wheat and barley. Most of the food was from those two grains, plus vegetables, and olive oil, and milk from sheep and cows. Today, people only want to go to the shop and buy the prepared products”.*	66
*Economic and inequality crisis*		
Increasing cash income dependence	*“Farming is not good anymore. Before, people used to make all of their food from the land—olives, almonds, taking care of the sheep, growing grain. Now people need real income, to cover all the needs”*	45
Greater market reliance and price hikes	*“[…] life was so hard and tough, but not expensive at all. […]. I just went to the market once a month, and did not spend much because I bought only small things—soap, spices […]. Everything is available now, but the products are getting really expensive—year by year”.*	49
Enhanced transport and cross-scale connectivity	*“All the places are connected to one another […]. I just need to go to the road and stop a car […] but before, either you walked or you rode a donkey”.*	18
Declining social cohesion and support	*“*[In the past] *everyone was taking care of others. They shared the good and the hard times, the food and the hunger.* […]*. Now everything has changed.* […] *they are always busy with their phones, they don’t care about others, they don’t care about their parent”.*	54
*Socio-cultural value crisis*		
Expanding life ambitions and planning	*“When I was twenty years old, I still played as a young child. I only cared about the food and to sleep. Now people are very ambitious. They plan their life”.*	16
Weakened work ethic and impatience	*“The old generation worked hard and they were really patient. The opposite is true for the new generation. They don’t like to work. They always take things in a hurry”.*	17
Expanding job expectations	*“Young generation—are too lazy. They just complain all the time. […] everyone, of the young people want to become minister, or I don’t know what exactly. When I was young, I used to do everything. Farming, harvesting, helping other people. I never complained”.*	31
Eroding moral and religious believes	*“When people used to stick to the religion, and behaved good, and be good to each other […] they used to be strong. But people gave up—they don’t follow the Islam and the real message from the Koran. Everyone now is selfish, there is a lot of injustice”.*	54

**Table 2:** Key dimensions of respondents’ affective ties to land and home, as encapsuled in the Amazigh concept “*tamazirt”*

Dimensions	Illustrative quotations	IDs
Freedom	*“For me, land—tamazirt—for me it’s home. My life. Even if people struggle, I feel always this kind of freedom—when I get to the family land, walk around the farming places. […]. I am always attached to the ground”.*	75
Identity	*“I was born as a farmer. I grew up with dirty hands—I was always in the field. Look at them—even they get older—but the land is in my blood. I really feel said about this new part of my life. People don't care about the others, neither about their land. I would never forget what I got from this soil. I will farm, until I pass away”.*	33
Contentment	*“But it is still a good thing* [farming despite limited returns]*, to have a harvest from our land. That makes us feel better and happy”.*	26
Belonging	*“I love it [the land]. It’s quite obvious. It’s where I belong, where I was born, where I have family—it’s home”.*	73
Stability	*“Land is one of the important things. It used to be and it will stay [important]. The world is now getting destabilised—food security—so the farming is the first important activity for the whole human being”.*	69
Food security	*“If there is a place where no one farms, it will be a terrible situation. It's all about farming. If people stop farming, where will we get our food? Where will we get our vegetables from”?*	73
Predictability	*“I always want more; I always want a new land. […]. More land means more security. […]. Having more pieces of land always means having more almonds, more olives, you can sell. It means no worrying anymore what the next day will be hiding”.*	75
Pride	*“I would like to have apple trees, and start a business from it. I can also open a small shop to sell products and settle down. But until now I can’t. […]. I like Tamazirt—it’s my home, and I want to make it the best village, if I could.”*	59
Care	*“I really care about the village, and I hope that one day everybody understands one another, and works together—forget about the politics problems, or land issues, and work all for the good of Tamazirt”.*	57
Commitment	*“I like farming, and I would like to do it. Especially apple and carob trees. I think it’s the future for this land. I fight for tamazirt, and I am always happy to come back and I feel sad when I have to go* [to work seasonally in Agadir]*”.*	57
Stewardship	*“Even if I am really old, I will go farming, growing my seeds, and harvesting. It’s a blessing from God. We should appreciate it”.*	18

## Results

### Spatial mobility patterns and proximate causes

Spatial mobility has featured in livelihood trajectories throughout the study area’s history and was ubiquitous at the time of our study. Senior respondents, aged 60+, recalled how they had once settled in the region to establish their families in a water-rich, agriculturally promising region. Yet, even in the 1960s some had found seasonal farm work in Souss. However, labour migration remained limited in subsequent decades, until farmers began engaging in off-farm work in the 1990s, when employment opportunities on urban building sites and in factories increased. A further step change followed in the 2000s, as *“the Moroccan economy grew up”,* and employment surged *“but in the city, not the countryside” *(Resp. 33).

### Domestic and international out-migration

The most commonly reported mobility pattern in the present was return labour migration to urban centres and commercial farms (Fig. 3). *“There are less* [local] *job offers than people who look for work”,* reasoned one respondent (Resp. 20) regarding the proximate causes of this mobility trend.

Men, especially those in their late 20s to early 40s often engaged in successive gigs in Morocco’s informal economy, as craftsmen, in tourism, construction, factories, or on farms near Agadir and Béni Mellal. Respondents perceived such migration as reasonable, with some expressing that the only alternative for young people in the countryside was *“taking drugs and sleeping the whole day”* (Resp. 70). Cities afforded greater independence and work to support their families. Employment in the formal economy was rare, in contrast to other proximate mobility causes, such as family formation after marriage, enrolment in locally inaccessible educational programs, and entrepreneurship (Fig. 4).

International out-migration was directed towards France, Italy, Algeria, Spain, Niger, and the United Arab Emirates, typically long-term, and reliant on social networks. Good salaries were realised in Algeria, for instance, where Moroccan construction design skills were in high demand. Others worked in the telecommunications, security, and electricity sectors of their destination countries. Often, they indicated to have worked under precarious circumstances, before their status eventually became legalised. *“I did not like that they took my passport. I am a free man”*, exclaimed a returnee (Resp. 69). Female international migration was seldomly reported, and was associated with marriage, when it occurred.

#### In-migration and rural flight

Four proximate causes of in-migration were reported: (i) family formation and reestablishment close to public services and city-jobs, (ii) engagement in seasonal work, (iii) retirement, (iv) and investments to start a business (Fig. 1a).

Before the introduction of regular school buses, the establishment of a rural school, and the upgrading of a secondary road that now reliably links communes throughout the mountains, in-migrants had settled in villages near Demnate, to (re)establish their families close to hospitals, schools, and city jobs. Land in these villages was cheaper than in the city, yet still close enough for commuting. As one respondent explained: *“My father was saving money for years, to settle down here”* (Resp. 55), emphasising that newcomers from biophysically harsher mountain environments were rarely wealthy. Another respondent felt that *“nowadays, the village is mixed”* (Resp. 48), explaining that established residents had sold plots to in-migrating families, using the profits to pay off loans or build new homes.

The second cause for in-migration was seasonal employment in the region’s modest tourism industry, which COVID-19 nearly halted. The third cause was a wish to return from abroad, to one’s place of origin upon retirement. And the fourth, respondents’ intention to invest, to commence their own business activities in the study region.

In addition to the peri-urbanisation dynamic caused by in-migration, as outlined above, a strong rural flight trend also emerged (Fig. 1b). Woman, in particular, settled in Demnate upon marriage. However, even from this urban centre *“people prefer to move or are forced to move*” to larger towns, explained one respondent, because *“there are few jobs to do”* (Resp. 8). Localised mobility, without residential shifts, was linked to the sale of agricultural crops, secondary school commutes, or residents’ employment.

#### Immobility

Voluntary immobility was common among many senior residents who had once made their living through traditional, subsistence-oriented farming. Nowadays, most relied on remittances from siblings or younger relatives.

Involuntary immobility affected older woman and those with caregiving duties for small children and for sick or disabled family members. *“Woman here eat, and sleep, and do the housekeeping”,* said one respondent (Resp. 31). *“They cannot do much, because there are no jobs for them”*. A young man wished for his sisters to become well-educated and financially independent, to avoid his mothers’ fate, who had been *“struggling a lot”* (Resp. 8). Respondents shared that the role of women was slowly changing, *“nowadays, more girls can go to school”* (Resp. 9). Yet, the effects of limited educational opportunities for women may take generations to fully fade. *“I would like to know how to read and write”*, lamented a woman with school-aged children (Resp. 32). *“If I only was educated, I could help them with their homework, and guide them in their studies”.* Another immobility cause was expectations to take on the family tradition of working in agriculture, particularly extended towards older siblings or only children. *“My father wanted me to stay, and farm, and take care of the sheep, as he used to do”,* explained one respondent (Resp. 56).

### Underlying causes of mobility and land-abandonment

In addition to the above-described proximate causes, respondents identified a broad range of social-ecological change dynamics that had occurred in the study area since the early 2000s (Table 1). The interplay and reinforcing effects of these change dimensions, which we have grouped into three compounding crises for greater clarity, although their boundaries are fluid, were seen as the underlying causes of residents’ mobility and land abandonment decisions.

#### Climate and resource crisis

Regarding the climate- and resource-related change dimensions, respondents described how land access and biophysical farming conditions in the area had always been tough: much of the terrain was stony and rugged; land holdings were small and, due to partible inheritance, subject to progressive subdivision; the soils were less fertile than those in Morocco’s agricultural plains; and distances between homes and cropland parcels were sometimes considerable. Yet, senior respondents still recalled a time when they were content and got by with the food products that the land provided: The land was lush—*“water was just flowing to the fields”* (Resp. 33)—and crops, fodder, and livestock were abundant. Farmers worked hard, based on their parents’ traditional agroecological knowledge and practices, but without agro-industrial inputs.

The situation shifted, in part, as a result of increasing water scarcity caused by drought and the proliferation of wells for groundwater irrigation. *“The drought does not help people to farm”,* explained one respondent (Resp. 65). *“I used to do it as my main job; now I have to work in different places”*. Livestock rearing, as well as crop cultivation, faced abandonment: *“His grandfather used to be the main shepherd”,* said one respondent about another (Resp. 24). Now, *“if they want a sheep for the Eid celebration, they need to buy it”*.

#### Economic and inequality crisis

Concerning the economic and inequality-enhancing change dimensions, respondents recalled a time, in the not-so-distant past, when life was risky and public services out of reach: *“Some children could die. Especially, if they got stuck in the snow, in the mountains, while they were taking care of the sheep”*, said one respondent (Resp. 54). *“We did suffer a lot”*, recalled another. *“A woman gave birth to the child into the field”* (Resp. 27).

In the present, many respondents experienced an economic squeeze that put their household under pressure: *“The poor people are suffering from the high costs of living”* explained one (Resp. 19), who no longer raised sheep or chicken due to a lack of money to feed them. Families found that their fixed expenses had steeply risen, as they gained access to public services, energy, water, telecommunications, and transport infrastructure, alongside increasing market integration. *“Life was hard before, yes”* exclaimed one respondent (Resp. 49).* “Now everything is provided, but you need more money to have all your needs. It’s all about money. If you do not have it, you cannot even eat”.*

Adding to this pressure were high and rising costs for agricultural inputs, including seeds, fertilisers, machinery, and farm labour. Cash-poor households found these hard to muster at the start of the growing season, and often failed to make them up again at harvest time:* “*[…] *most farmers loose more than they get”,* said one respondent (Resp. 21). As a result, subsistence farming no longer sufficed to cover living costs, leaving ever more people dependant on off-farm work, which was hard to find locally.

Two respondents poignantly summarised the growing economic divide and shifting power relations within communities: *“People were united* […] *no difference at all between rich and poor”*, recalled one (Resp. 27). But *“nowadays, money is power”*, exclaimed the other, *“and if you are poor, you don’t have any power”* (Resp. 33). Economically more affluent households could build wells, purchase land, and pay for labour and material farm inputs. Less fortunate respondents bemoaned a lack of support from state institutions, such as subsidies, affordable loans or adult education and employment programs, which may have helped them to cope with, and buffer against lost agricultural livelihood prospects.

#### Socio-cultural value crisis

Focussing on the socio-cultural change dimensions, respondents’ spoke of shifting values and lifestyle aspirations that divided generations, and can be seen as a third main root of local residents’ mobility and land abandonment decisions: *“It’s another age—technology has affected people—they forgot about their identities”*, said one respondent, concerned about young people’s TV habits (Resp. 52). He feared that this new source of inspiration led them to forget about the meaning of the land. Another expressed his disdain for younger residents’ desire to own big houses, cars, phones, and expensive clothes: *“Nowadays, people don’t care about morals and respect, only about money and appearance. We call it* […] *the generation of speed time—it is only about how much you can get”* (Resp. 33). Others explained that *“the village used to live in harmony”*, when people’s relationship with the land and one another were strong (Resp. 18), but now young people lacked contentment, faith, generosity, and were ignorant towards the finitude of life.

Interviewees also worried about young people’s individualistic outlook and uncertain life prospects: *“Some young people nowadays just stay at home and consume a lot of drugs. They don’t care about their future and their family”*, said one respondent (Resp. 31). Another disagreed, believing that younger people were full of ambition and dreams but lacked the patience to achieve them: *“*[…] *everything needs time to be reached”*, she said, *“If you can fly you can fly, but if you can’t you can also walk”* (Resp. 66). A third respondent seemed to have lost all hope for farming futures in the region: *“Everyone looks only for money; the land has no value anymore”,* he said (Resp. 39). The reflections of a young respondent seemed to prove him right: *“I would rather have a small income than taking the risk of farming, and only depend on the rain”* (Resp. 75).

#### Migrations bidirectional effects

Although out-migration was a key crises response, it was rarely seen as a main land abandonment cause. *“Migration is not affecting the farming, but the drought is”,* exclaimed one respondent (Resp. 48). However, spatial mobility was perceived as exacerbating the crises complex that drove land abandonment decisions in several ways. Migration could push financially disadvantaged households into debt: *“They got credits from other people, and say my children will support me, to give it back. The children migrate, and come back with empty pockets. So, the only way to pay the debt is to sell a part of land”,* said one respondent (Resp. 53). Seasonal migration also reinforced a local farm labour shortage that high salary expectations from young residents, unwilling to provide free labour to extended family, drove. Migration further reinforced value and aspiration shifts through exposure to city life. A younger respondent, whose family had moved to a nearby city, elaborated: *“We grew up in an atmosphere, where farming is not a big deal”* (Resp. 30).

Conversely, migration enabled others to stay in the village or eventually return: *“Now I can grow vegetables that I can sell”* exclaimed one respondent (Resp. 71), whose land investments were made possible through remittances from his brother. Another explained how past migration enabled him to return to farming: *“I have worked in* [commercial] *farms for years* […] *that was my source income, to build this house, and to buy the land for farming”* (Resp. 53). One respondent aptly summarised how migration simultaneously drove people away from, and back to the land: *“My dream or goal now is to go to Algeria, and do like my cousin and his friend did. Make my own money, take care of the family, and then I could think a little about farming. If I had money, I can build a well, I can irrigate the olives and the almond, and help other people in the village”* (Resp. 55).

### Household constellations and intersectional characteristics

**Fig. 5:**
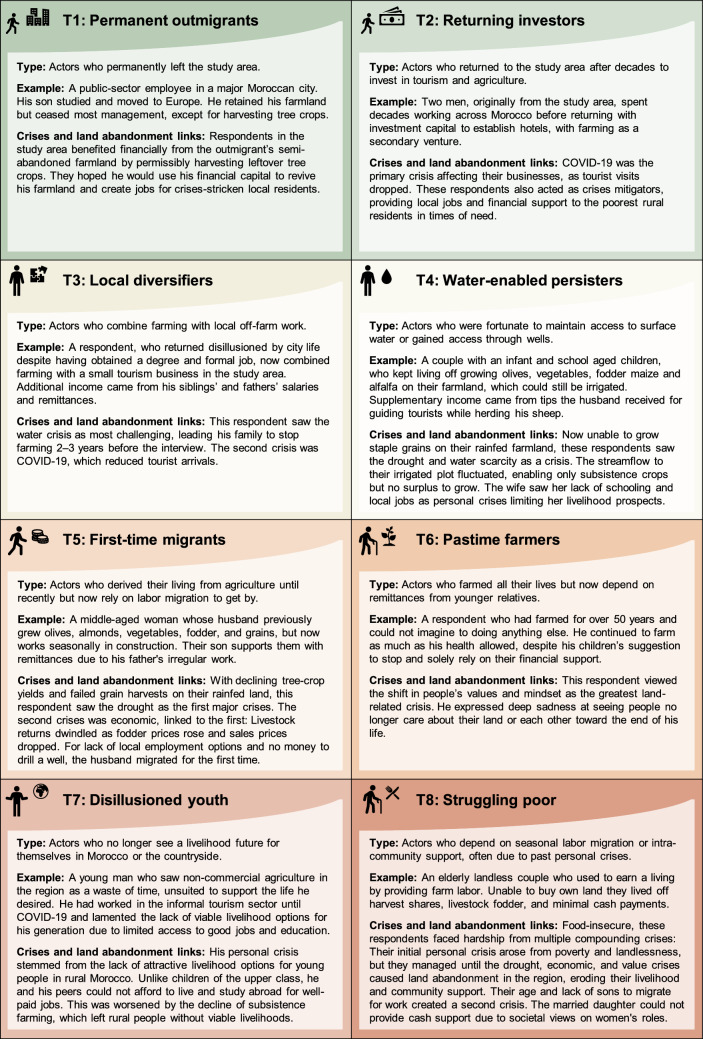
Typology illustrating the diverse mobility and land abandonment decisions of eight typical actor types, shaped by varied household constellations and intersectional characteristics, within a landscape of compounding crises

The landscape of compounding crises that residents in the study area faced left respondents questioning to what extent agricultural livelihood activities remained viable and meaningful to pursue. Their decisions regarding this question varied widely, mediated by their personal crisis experiences, intersectional characteristics, and specific roles and responsibilities in relation to other household and extended family members. We identified eight distinct actor types (Fig. 5).

Despite these profound differences among actors’ crises experiences, three cross-cutting narratives about spatial mobility and land abandonment decisions stood out (Table S3):

*“Wasting time”* encompassed notions of agriculture being no longer worthwhile, unless one possessed investment capital to transition from traditional to commercial farming. After all, *“this generation are lucky—they can get to the schools, study, have better jobs”*, said one respondent (Resp. 49).

*“Forced to move”* encapsulated notions about migration—with often accompanying land abandonment—as the only option to sustain a living for economically less affluent respondents: *“Migration is the only way to run away from poverty”,* exclaimed one of them (Resp. 8). Another explained how his aspirations would have remained unattainable, otherwise: “*I stopped farming, because I needed money to build my house*” he explained, *“so I sold my land and kept working as a construction worker for the rest of my life”* (Resp. 23).

*“Nothing to do”* captured women’s notions about being stuck in the countryside with very limited prospects to earn a living for themselves. Many older ones had once shouldered substantial parts of their households’ agricultural tasks. Yet, with the creeping end of subsistence farming, few local options remained to obtain a salary or engage in commercial tasks. In particular, older women, without husbands or sons to provide for them found themselves slipping into personal crisis: *“Life is so challenging, especially for women. They cannot find jobs. They cannot make an income. They just depend on the mercy of the life and their husband”,* said one (Resp. 26).

### Future aspirations and affective ties to land

Immobile respondents were not the only interviewees talking about ‘wasted time’ in their lives. While this notion was linked to agricultural land abandonment in one of the narratives outlined above, it also resurfaced in conversation with return migrants, though with a different focus. Some of the latter had become disillusioned with labour migration, and narratively framed it as a waste of time; just as their counterparts had done with respect to agricultural livelihoods: *“*[…] *working for those people* [in the tourism sector] *is like being a slave. No good salary, no future, only wasting time” (*Resp. 68), explained one respondent. Another elaborated: *“I have been migrating in different cities.* […]. *And many years have passed for me without any progress. It’s like only moving from one place to another, without any result to mention”* (Resp. 44).

Several respondents with these experiences shared a desire to finally settle in their rural birthplace for good. “*The city is too crowded; the village provides the secure atmosphere. So calm.* […]. *You can leave the kids out—not like in the city”*, explained one respondent* (*Resp. 75). A female interviewee shared similar sentiments, expressing a wish to move from Demnate to the countryside, where people were nicer, the lifestyle calmer, and residential rents lower, thus freeing up cash for food expenses.

Common to these accounts was that they appeared to reflect interviewees’ persistent and deep-rooted connection to their “tamazirt”. This concept, an Amazigh term for land and home, emerged as a shared and multi-faceted anchor point in interviewees’ narratives about their affective ties to and future aspirations for the study region’s landscapes (Table 2). For instance, highlighted feelings included freedom and contentment that respondents experienced through connecting with and working the land, as well as a sense of care for one’s community and its residents. Respondents’ future aspirations ranged from continued farming until the end of one’s life, over land accumulation for securing a predictable livelihood, to starting a commercial tree-growing business warranting pride.

## Discussion

**Fig. 6:**
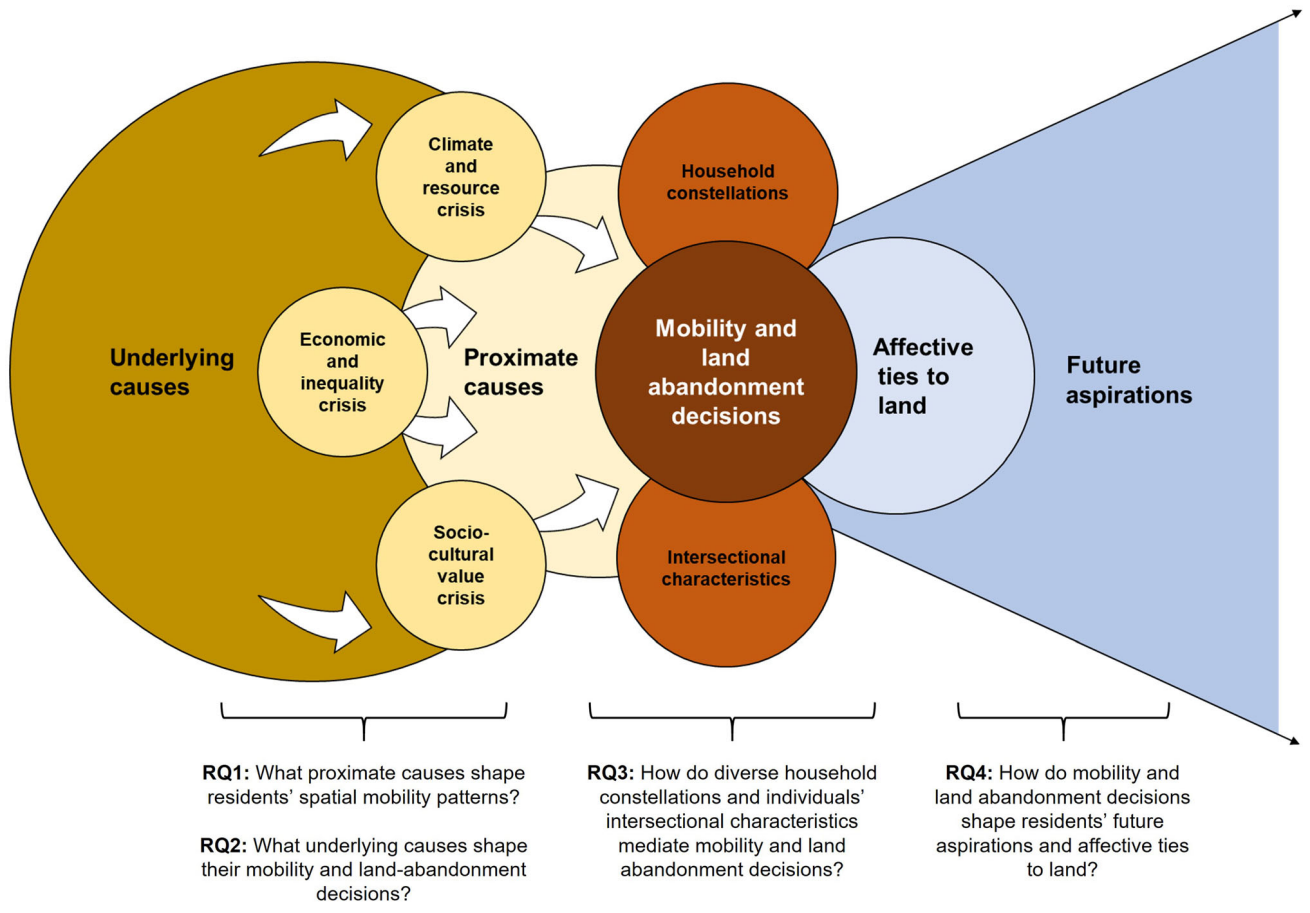
Conceptual representation of how residents’ mobility and land abandonment decisions are shaped by proximate and underlying causes, mediated by specific household constellations and intersectional characteristics, and how they shape affective ties to land and future aspirations in the study region

Our research objective was to appraise the perceived importance of water insecurity and a severe drought, relative to other crises, as causes of rural people’s spatial mobility and land abandonment decisions in Morocco’s High Atlas. In this regard, our study highlights that, due to diverse causes, both mobility and land abandonment are integral to rural people’s everyday lives. Yet, far from everyone is on the move, and our analysis shows that farmers’ decisions to stay or migrate and to reduce or halt their agricultural activities are best understood as being rooted in diverse social-ecological change dynamics, which we grouped into three compounding crises (Fig. 6). Individuals’ differentiated household constellations and intersectional characteristics mediate their decision making, which both shapes and is shaped by their future aspirations and affective ties to land.

### Compounding crises cause spatial mobility and land abandonment decisions in Morocco’s High Atlas

Regarding our first research question on spatial mobility patterns and their proximate causes, we found domestic labour migration to commercial agricultural regions and urban centres to be most common (see Figs. 3 and 4). In addition, international migration and mobility for family and educational reasons also played a role. These insights resonate with recent findings from studies in Morocco’s Al Houz province, in Souss-Massa, and the Assaragh Basin, which likewise highlight seasonal labour migration to Morocco’s large cities—rather than international migration—as an increasingly common livelihood pursuit (Boselli et al. 2020; Ferreira Fernandes et al. 2024; Boubou et al. 2025). In line with our analysis, which identified university and school attendance as proximate drivers of young respondents’ mobility decisions, Ferreira Fernandes et al. (2024) also recognised better educational opportunities as a secondary motivation for migration. Focussed on the base of the High Atlas our study complements a recent country-level assessment of Morocco’s migration complex (Berriane et al. 2021) by providing detailed insights into migrant flows and their proximate causes from a previously unmapped area.

Our second research question examined the underlying causes, including crises experiences (see Table 1), that influenced respondents’ spatial mobility and land abandonment decisions in greater depth. One central finding is that water scarcity played a key role in residents’ decision-making (see Section “Climate and resource crisis”), in line with evidence from Amazigh communities in Morocco’s Al Haouz province (Boubou et al. 2025; Woodmansee et al. 2025) and various international land abandonment studies (Subedi et al. 2022). However, in contrast to Woodmansee et al. study (2025) our respondents often cited drought and water insecurity as secondary challenges that exacerbated an already complex crises situation. They did not see these factors as the primary cause of their land abandonment decision. Instead, they attributed the roots of their challenging living conditions to a fundamental crisis of their formerly subsistence-oriented livelihoods, which had been destabilised by their households’ increasing reliance on paid public services and largely completed integration into commercial food and agricultural markets, making them dependent on cash (see Section “Economic and inequality crisis"). Our findings thus reinforce the argument that rising social disparities and uneven economic growth drive rural out-migration from Morocco’s interior (Berriane et al. 2021), while also illustrating how this national trend manifests locally in Amazigh communities.

Our study further highlights the role of shifting values, cultural norms, and lifestyle aspirations as underlying causes of mobility and land abandonment decisions (c.f. Berriane et al. 2021) (see Section “Socio-cultural value crisis”). Sentiments about young people’s intensified materialism, haste in achieving life aspirations, and respondents’ narratives of “wasting time”, “being forced to move”, and “having nothing to do” resonate with findings from Spain (Quintas-Soriano et al. 2023). They also signal a crisis of confidence regarding the attainability of a fulfilling rural life among respondents that subsumes both material, land-related, and non-material concerns. Sectoral measures alone may thus not suffice to address the crises nexus that drives mobility and land-abandonment decisions among High Atlas residents. Policy initiatives to solve the country’s water crisis, such as Morocco’s national water management strategy, are crucial for easing pressure on rural households. Yet, analysts warn that cross-sectoral coordination and pre-emptive actions to address social, economic, and environmental externalities remain insufficient in Morocco’s policy frameworks (Meir et al. 2022). This is concerning, as a recent flagship assessment on the interlinkages among global biodiversity, water, food, health, and climate concerns highlights the importance of nexus approaches to effectively address compounding crises (IPBES 2024).

### Multi-locality and intersectional traits shape individuals’ crises experiences

Addressing our third research question, we explored how different household constellations and respondents’ intersectional traits mediate their crises experiences, and associated mobility and land abandonment decisions (see Section "Underlying causes of mobility and land-abandonment”). A key contribution in this regard is our eight-category actor typology (see Fig. 5), which adds analytical depth to a set of previously identified livelihood strategies (Woodmansee et al. 2025) that drought-stressed households embrace. Our typology helps address a persistent knowledge gap in the literature on intra-household dynamics in environmental migration decision-making (Zickgraf et al. 2022) by highlighting how the mobility of certain household members—across different household constellations—enables others to either continue farming or advance alternative land-reliant livelihood activities, even amidst multiple compounding crises.

Our analysis further underscores that involuntary immobility was fairly common among less affluent, female, and older residents, consistent with findings from research on migration barriers in drought contexts, such as southern Morocco and Bangladesh (Afifi et al. 2016; Ferreira Fernandes et al. 2024). For others, immobility and even land investments were a deliberate choice upon their return from previous migrations that had enhanced their relative prosperity, thereby making them less vulnerable to the impacts of contemporary crises. Taken together, these findings highlight the importance of migration as a crises buffer and underscore relational perspectives and the climate mobilities premise that rural people’s abilities and decisions to migrate or stay are often interlinked in time and space (Boas et al. 2022; Natarajan et al. 2022; Phongsiri et al. 2023).

The rising multi-locality of many households, and the apparent but incomplete abandonment of former farmland, result from rural residents’ broadening of their livelihoods both through spatial mobility and by engaging in local off-farm activities. This is not a uniquely Moroccan phenomenon (Natarajan et al. 2022; Subedi et al. 2022). For instance, parallels can be drawn with southern Europe and Asia, where these dynamics have been studied in greater depth, though not conclusively (Dolton-Thornton 2021; Ojha et al. 2022; Phongsiri et al. 2023). Exploring insights from these contexts could be valuable for drawing lessons on how to more effectively recognise mobility and livelihood diversification patterns within Moroccan agriculture and rural development programs and government strategies. For example, the Moroccan Green Generation Strategy could go further in addressing rural poverty by accounting for households’ increasing split of efforts between migration, agriculture, and local non-farm work, emphasising interventions that enhance local labour absorption, especially during droughts and off-peak seasons (Chiarella et al. 2023). Additional focus could be placed on how the most vulnerable, who lack resources to become agricultural entrepreneurs, can be supported to live dignified lives, and on how to better engage woman whose societal roles and aspirations are steadily shifting, even in rural areas. Finally, interventions could leverage the social and financial capital of more prosperous return migrants or remitters in cities, those able to invest in realising Moroccan policy makers’ ambition to develop a new agricultural middle class.

### Tamazirt: a promising anchor for land-futures thinking

To address our fourth research question, we appraised respondents’ land-related future aspiration and their affective ties to land (see Section “Future aspirations and affective ties to land”). One crucial insight in this regard was that a non-negligible number of interviewees saw their future in the High Atlas, rather than Morocco’s major cities. They perceived their identity, heritage, and well-being as deeply intertwined with the region’s landscapes—as expressed through the multi-faceted emotions and aspirations, which they narratively associated with the Amazigh concept *tamazirt* (see Table 2). This insight—that “land is home” and culturally imbued—echoes land system research that foregrounds marginalised communities’ justice concerns (Meyfroidt et al. 2022), and the centrality of culture in land-use decision-making (Hodel et al. 2024). The former resonates with our study context, as Amazigh communities’ land and natural resource rights face growing privatisation and commercialisation pressure, under the guise of new land laws and sectoral investment programmes, such as the Green Generation Strategy (IWGIA 2024). The latter encapsule two oppositional dynamics that we observed in our study: The role that value crises may play in destabilising long-established land-use systems, and the enabling potential of cultural values and practices for the persistent stewardship and revitalisation of traditionally managed lands (Hodel et al. 2024).

The continued importance of *tamazirt* that our analysis revealed suggests that this Amazigh concept could serve as an indigenous anchor point for reflections on how to revitalise Morocco’s abandoned farmlands. Land system studies on reutilisation pathways remain limited (Subedi et al. 2022), despite many conceivable path (e.g. agricultural intensification, ecological restoration, or land-use diversification) that should be weighed for their respective merits and trade-offs in different social-ecological settings (Munroe et al. 2013). In Morocco, such research could be advanced in conjunction with the further development of rural development policies, such as the Green Generation Strategy. This would imply involving governance actors and implicated landscape residents in co-learning processes, critical dialogue, and decision-making for desirable and just land-use futures. The grounding of such activities in Amazigh language and land-stewardship traditions would fit our respondents’ aspiration to promote culturally-grounded culinary and tourism experiences to revitalise their regions’ landscapes and secure local livelihoods. Actors aiming to initiate such revitalisation paths could draw on the results of a recent study on communities’ biocultural heritage in the study area (Mobarak et al. 2025), instructive experiences of numerous but often overlooked local development initiatives in other world regions (Londres et al. 2023), and on a suite of systematically-identified approaches to facilitate indigenous futures thinking (Cheok et al. 2025).

## Conclusion

Increasingly complex crises challenge dryland farmers’ mobility and land abandonment decisions. In this study, we traced how rural residents navigate migrating and land-abandonment in a drought-affected region of Morocco’s High Atlas. Our findings highlight that fundamental shifts in communities’ economic and socio-cultural fabric, disparate household circumstances, and personal attributes create a landscape of compounding crises that water scarcity escalates. Individuals’ crisis experiences vary widely, with some remaining immobile, others returning, or hoping to stay. This underscores a critical need to create pathways towards dignified livelihoods and revitalised High Atlas landscapes.

Our study highlights three learning points in this regard: The first is that migration and land abandonment are common, though far from always desired, realities for dryland farmers whose traditional livelihoods become undermined. Another key point is that nexus thinking is crucial to address their predicament, holding decision-makers accountable for translating the synergistic response options from the IPBES nexus assessment and enhanced social safeguards for the most vulnerable segments of society into Moroccan policy frameworks. Finally, *tamazirt* and similar indigenous concepts and traditions should be championed as a foundation for locally rooted development, reigniting social cohesion, attracting tourists, and engaging support and investment from well-endowed former residents.

Future research could explore perceptions and life experiences of long-term migrants, new urbanites, and Morocco’s diaspora. Our study did not capture their views, as its entry point was firmly rural. Assessments of their mobility motives, ties to origin communities, and land-related future aspirations may reveal new perspectives for revitalising Morocco’s abandoned farmlands. The striking peri-urbanisation and rural flight dynamics that our appraisal captured also warrant further study. Tracing households’ residential shifts along a gradient from remote villages, to peri-urbanising settlements, and expanding cities may reveal persistent livelihood challenges behind individuals’ relocation decisions, and offer critical foresight into demand for municipal infrastructure, spatial planning, and land value regulation, to prevent inequality-driven displacement and urban sprawl.

## Supplementary Information

Below is the link to the electronic supplementary material.

Supplementary file1 (PDF 147 KB)

## Data Availability

The data that have been used are confidential.

## Declarations

### Use of generative AI tools

ChatGPT 4.0 was used for language improvement in writing this article.
