# Supplementary material for: Compounding social-ecological crises drive spatial mobility and land abandonment in Morocco’s High Atlas
Source: Ambio. 2025 Dec 19;55(7):1555–71. doi: 10.1007/s13280-025-02323-5 (PMC13230355; doi:10.1007/s13280-025-02323-5)
Supplement: Supplementary file 1 — Supplementary file1 (PDF 147 KB) [file 13280_2025_2323_MOESM1_ESM.pdf]

Ambio

Supplementary Information

This Supplementary Information has not been peer reviewed.

Title: Compounding social-ecological crises drive spatial mobility and land abandonment in Morocco’s High Atlas

Table S1. Respondent characteristics.

Respondent characteristics	Share of respondents [%]
Gender	
Male	75
Female	25
Age group	
Younger than 35	21
35 - 60	32
Older than 60	23
Exact age not recorded	24
Own mobility history	
Yes	64
No	24
Not recorded	12
Current occupation (main)	
Agricultural sector	27
Other sectors	41
Woman engaged in farming and domestic work	15
Retired	9
Unemployed	3
Not recorded	5

Table S2. Iteratively developed interview themes and questions.

**Iteratively developed interview themes and questions**
Respondent, household, and family information<br>• Respondent's name and place of residence?<br>• Age and main occupation of the respondent?<br>• Household size?<br>• Main occupation of other household members?
(Im)mobility experiences and trajectories<br>• Respondent's arrival at the current place of residence?<br>• Reasons for and timing of past (im)mobility decisions and events?<br>• Current mobility patterns/temporary stays away from the place of residence of household members<br>• Experiences made and reflections on previous mobility events<br>• Mobility decisions and experiences of previous household members, now living elsewhere<br>• Maintained links to mobile household and other family members, e.g., remittances<br>• Perceived reasons for (im)mobility decisions of community members<br>• Links between (im)mobility decisions and occupational opportunities in the study region
Farming experiences, trajectories, and land-use decisions<br>• Respondent's previous and past engagement in farming<br>• Previous and past engagement in farming of other household members<br>• Junctures in farming engagement, including underlying reasons<br>• Links between own and community members' (im)mobility decisions and farming in the study region<br>• Reflections on agricultural landscape and livelihood changes over the respondent's life course<br>• Experiences with and reflections on the Green Morocco Plan and associated interventions<br>• Respondent's affective ties to farming and the meaning of "tamazirt"
Water scarcity, drought, and links to (im)mobility decisions<br>• Experiences with drought, water scarcity, and perceived climatic changes in the region<br>• Perceived reasons for and impact of water scarcity and drought on farms and livelihoods<br>• Perceived links between water scarcity, drought, and own and community members' land-use and (im)mobility decisions
Future aspirations<br>• Respondent's aspirations regarding future (im)mobility and place(s) of residence<br>• Respondent's aspirations regarding future occupations, farming engagement, and agricultural development in the region<br>• Experiences with and aspirations regarding future tourism development in the region

Table S3. Key narratives on respondents' crises-driven mobility and land abandonment decisions, illustrated with representative quotations.

Narratives	Illustrative quotations	IDs
"Wasting time"	*"The land here does not really help.* […] *to start commercial farming, you need a lot of money, and big land. Otherwise, it's just a waste of time and money."*	30
	"The young people left the village, and if you try to speak with them in the village interest – maybe in creating a new society – they would answer: 'There is nothing to do there, you are just wasting your time.'"	57
	"Farming is a wasting of time. Either you have a big farm, and you harvest a lof of quantities, and you do it as a commercial work – or you just sell the land if someone would like to buy it, and leave this place for once. What's the point of farming – if it does not provide money?"	68
"Forced to move"	*"More young people are leaving. But they are forced to do it, because they need to make money."*	17
	"I work normally in a clothes factory. It is not better [than farming] *but there is no more option. My mum and small sister depend on me."*	46
	"If I found a job here, I would stay near to my family. But it is the only way to do it – to make my living. Migration does not meet my aspirations, but at least I can buy some food for my family and help a little bit."	59
	"If it was an option for me, I would really like to go back to the village and settle there. Built a well, buy a tractor. If it was an option, I would like to farm. Selling vegetables in the souk. The idea of being a farmer that I have is deep inside. But as I can't, I am forced to migrate and live my life somewhere else."	57
"Nothing to do"	*"For women, they are only housewives. There is not much to do here. No cooperatives, and associations. I used to gather some thyme and give it to my husband to sell it. But it does not provide an income."*	70
	I am the one who is doing the harvesting of olives and similar works […]. I am also the one who is always taking care of the sheep. […]. My husband sometime sells some, during Eid time. For me, there is nothing else to do in the village. There is no cooperative or association or something, where women can get some income."	43
	"Here the women are kind of lost. They don't do nothing, and they know almost nothing. They are not trained somewhere. Only cooking and raising children."	32
